# Development of an experimental model using cold stress to assess the pathogenicity of two Moroccan AI H9N2 isolates from 2016 and 2022 in commercial broiler chickens

**DOI:** 10.1371/journal.pone.0320666

**Published:** 2025-04-04

**Authors:** Oumayma Arbani, Mariette F. Ducatez, Mireille Kadja-Wonou, Faiçal Salamat, Faouzi Kichou, Mohamed El Houadfi, Siham Fellahi

**Affiliations:** 1 Department of Veterinary Pathology and Public Health, Institut Agronomique et Vétérinaire Hassan II. Rabat BP, Morocco; 2 IHAP, Toulouse University, INRAE, ENVT, Toulouse, France; 3 Department of Public Health-Environment, Ecole Inter-Etats des Sciences et Médecine Vétérinaires (EISMV) of Dakar, BP Dakar, Sénégal; Cairo University, EGYPT

## Abstract

Since 2016, low pathogenic avian influenza virus (LPAIV) H9N2 became a major issue for poultry production in Morocco. Even though the agent was classified as low pathogenic, AI H9N2 cause significant economic losses, particularly during co-infections. Experimentally, it has been difficult to reproduce the clinical picture without appealing other viral or bacterial pathogens. Our study was carried out to evaluate a new challenge model using cold stress in commercial broilers infected with two Moroccan H9N2 viruses isolated in 2016 and 2022. One hundred twenty day-old chicks were divided into four groups: A, B, and C exposed to cold stress, and D was kept as negative control. At 21 days of age, Groups A and B were challenged by oculo-nasal route with 10^7^ EID_50_ of H9N2 strains, isolated respectively during 2016 and 2022. Meanwhile, chicks of group C were exposed to only cold stress. The assessment of body weight gain, clinical signs, lesions, mortality, and oropharyngeal viral shedding was monitored for 15 days post-challenge. Results showed that cold stress exacerbated H9N2 clinical signs, allowing us to establish a scoring system and to validate the challenge model without co-infections. Gross and microscopic lesions, induced by the virus primarily in the respiratory tract, peaked at 5 dpi and significantly decreased at 15 dpi. Group B harbored the highest viral loads with viral shedding persisting beyond 11 dpi in both groups. This study demonstrates a clear clinical difference among the two isolates; A/chicken/Morocco/178-2/2022(H9N2) showed a significant increase in virulence compared to the firstly isolate A/chicken/Morocco/SF1/2016(H9N2). The novel H9N2 challenge model using cold stress will contribute to a better understanding of LPAI pathogenesis and epidemiology and allow for research closer to the field.

## Introduction

Low Pathogenic Avian Influenza Virus (LPAIV) H9N2 subtype, is one of the most widespread avian influenza viruses worldwide [1]. This virus, which was first identified in turkeys in Wisconsin, USA, in 1966 (A/turkey/Wisconsin/1/1966(H9N2)) [2], has spread rapidly throughout Asia, Africa, the Middle East and parts of Europe. It poses a significant threat to both human and animal health due to its ability to adapt to mammalian-like receptors [3–5]. With its contribution as a gene material donor to other severe zoonotic viruses, notably the highly pathogenic H5Nx viruses of the Goose/Guangdong/1996 lineage and the H7N9 viruses of the Anhui/1/13 lineage, it has led to fatal zoonotic diseases [6,7].

In Morocco, since its first detection by El Houadfi et al, in 2016 [8], AI H9N2 has continued to spread widely across the country, causing significant economic losses. This endemic circulation has led to newly detected mutations in the virus genome, resulting in a notable increase in its virulence in the field, despite the implementation of vaccination programs [9,10].

Under field conditions, LPAI H9N2 infection has consistently been associated with respiratory signs (e.g., coughing, sneezing, and rales), high mortality rates (ranging from 5% to 30% depending on co-infections and management practices), a significant drop in egg production (up to 60% in some cases), and a marked decrease in feed consumption [11–14]. These symptoms are often aggravated when the infection is associated with other pathogens or stressful field conditions; including respiratory viruses such as Newcastle disease virus, Infectious Bronchitis virus, and Infectious Laryngotracheitis virus [15–17], as well as, several different bacteria including Salmonella, E.coli, *Avibacterium paragallinarum*, *Ornithobacterium rhinotracheale* and *Staphylococcus aureus* [18–21]. The pathogenicity of LPAIV H9N2 strains have been investigated in numerous studies using avian models under experimental conditions to replicate the clinical and lesions observed in the field [12].

In domestic poultry, the most notable lesions in affected birds include congestive tracheitis, mild to severe pneumonia, and severe exudation leading to cast formation in the tracheal bifurcation, extending to the lower bronchi. Given this, when evaluating the pathogenicity of the virus under experimental conditions, reproducing the respiratory symptoms, lesions, and mortalities seen in the field is challenging and often failed without integrating other pathogens in the challenge model [12,22].

To better understand the disease severity observed in the field, we have developed a new challenge model using cold as a stress factor. Using this challenge model, we established a scoring system to quantify respiratory and ocular signs, as well as macroscopic and microscopic lesions. Additionally, we investigated the pathogenicity in broiler chickens of LPAIV H9N2 isolates collected from Moroccan poultry field: one from the first AI H9N2 cases in 2016 and another isolated from AI H9N2 outbreaks during 2022. This approach allows us to exacerbate the clinical signs and macroscopic lesions as observed under field conditions.

## Materials and methods

### Ethics statement

All animal research conducted in this study received ethical approval for sampling and animal manipulation from the institutional Ethical Committee for Animal Veterinary Science and Public health (CESASPV) under the number CESASPV_2022_A02. The protocol was applied in accordance with international standards cited in numerous scientific references and the OIE Manual (2015) titled “Manual of diagnostic tests and vaccines for terrestrial animals”[23].

### Experimental conditions and birds

Hundred twenty day-old commercial broiler chicks of the Ross 308-line breed (Aviagen, Spain) were purchased from a local hatchery and reared in the same condition until 36 days of age. At the start of the experiment, chicks were divided into four equal groups and housed separately in enclosures of 4m^2^ each (22°C, RH 60%, 20-hour lighting program [24,25]). Feeding and care were conducted in accordance with the approved guidelines by the Ethics Committee. All procedures of controlled and secure experimental environment were carried out within a negative-pressure Biosafety Level 2 room at Institut Agronomique et Vétérinaire Hassan II in Rabat, Morocco. Throughout the study, feed and water were available *ad libitum* and all biosecurity measures were strictly implemented.

All chicks possessed maternal antibodies against H9N2 influenza virus, detected using hemagglutination inhibition (HI) test conducted with 4 hemagglutinin units (4 HAU) in a V-shaped 96-well microplate, following the guidelines outlined in the World Organization for Animal Health (WOAH) manual [23].

In the hatchery, all chicks were vaccinated with Recombinant live-virus vaccine (HVT-IBDV-ND) against Marek’s disease virus, Infectious Bursal disease virus and Newcastle disease virus by sub-cutaneous route. During the experiment, the birds have received vaccines against infectious bronchitis (H120) at one day old by nebulization and at day 14 against Newcastle disease virus in drinking water.

### Challenge viruses

Avian influenza viruses used in this study were A/chicken/Morocco/SF1/2016(H9N2) (Genbank accession number for HA: **LT598512**) characterized by El Houadfi et al, (2016) [8] and A/chicken/Morocco/178-2/2022(H9N2) (Genbank accession number for HA: **OR592451**) provided by Arbani et al, (2023) [9]. Preparation and titration of viral stocks were conducted in 9 to 11-day-old chicken embryonated eggs following the WOAH protocol [23]. The median embryo infectious dose (EID_50_) was calculated using the methods reported by Reed and Muench, 1938 [26]. The viral stocks were diluted in a medium containing antibacterial antimycotic agents (Penicillin, streptomycin, and amphotericin B (100x)), to obtain a final titer of 10^7^ EID_50_/ml, resulting in a dose of 10^6.3^ EID_50_ per animal. This viral load is higher than the one observed in naturally infected birds, which range between 10^3^ to 10^5^ EID_50_/ml, depending on the stage of infection, host immune status, and co-infections [12,27,28]. The use of a higher approach viral load is a standard practice in experimental challenge studies to ensure uniform infection and to systematically evaluate pathogenicity under controlled conditions [29]. The harvested chorio-allantoic fluid was tested for hemagglutination and subjected to qRT-PCR using the primers and probe for detecting HA2 subunit of H9 subtypes as described by Monne et al. [30].

### Experimental trial design

**Fig 1:**
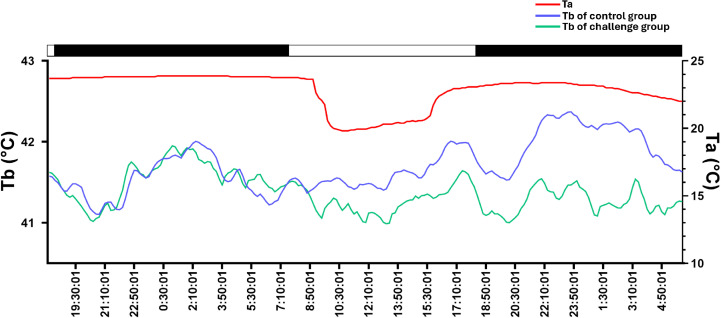
Experimental conditions during the cold stress exposure. Ta = Ambient temperature. Tb = Body temperature.

Divided groups were named from A to D. Groups A to C were exposed to 6 hours of cold stress (+14°C) 24 hours before the virulence challenge while group D (negative control) was kept at 22°C. To monitor body temperature, subcutaneous microchips were inserted in all birds. Temperature monitoring was conducted using data loggers (Thermochron, iButton, DS1922L, Dallas Maxim Integrated Products, Dallas Semi-Conductor). iButtons were programmed for continual recording of temperature with 16-bit resolution 0.0625°C every 10 min. The implantation site was selected based on previous validation studies in different species showing minimal migration and optimal reading consistency [31–34]. Prior to implantation, the area was cleaned with antiseptic solution and local anesthetic was administered (Local lidocaine 2% infiltration (0.1 ml)). Ibuttons were implanted subcutaneously in the dorsal thoracic region of each chicken in exposed groups using a sterile single-use applicator at a 45-degree angle, lateral to the spine. After the end of the experiment, Ibuttons were recovered and temperature reading was realized using the 1-Wire interface which is a device communicating with the computer. This interface uses a specific software, the One wire-viewer (Dallas Maxim Integrated Products), as presented in **Fig 1**.

At 21 days old, chicks from groups A and B were challenged by oculo-intranasal route with 0.2 mL of 10^7^ EID_50_/ml Moroccan H9N2 virus isolated in January 2016 (A/chicken/Morocco/SF1/2016) and in 2022 (A/chicken/Morocco/178-2/2022), respectively. Chicks from Group C were only exposed to cold stress and inoculated with phosphate-buffered saline (PBS) by the same route. Finally, group D contained the negative control flock, which were unexposed to cold and only inoculated with PBS via the same route.

The study employed a systematic euthanasia approach to evaluate the experimental model induced by cold stress. Ten birds from each experimental group were randomly selected for euthanasia at 5-day intervals throughout the post-infection period. Euthanasia was performed using sodium pentobarbital solution (300 mg/ml) administered via intracerebral injection. Throughout the 36 days of the experiment, daily monitoring of animal health, behavioral parameters as well as animal care and feeding protocols were conducted following the guidelines approved by the Ethics Committee.

The design of the study and experimental groups are summarized in Table 1.

**Table 1:** Experimental design used in broiler chickens.

Groups	Group A (n = 30)	Group B (n = 30)	Group C (n = 30)	Group D (n = 30)
Challenge strain	AIV isolated in 2016 **A/chicken** **/Morocco** **/SF1/2016** **(H9N2)**	AIV isolated in 2022 **A/chicken/Morocco** **/172-1/2022 (H9N2)**	Inoculated with PBS	Inoculated with PBS
Cold stress	Exposed 6 hours to cold stress (+14°C), 24 hours before challenge			Non-exposed to cold stress
Inoculation route	Oculo-nasal route at 21 days of age			

### Body weight gain

Body weights were recorded individually once a week for all groups until challenge. From the 2^nd^ day post-infection (dpi), the body weight of the experimental birds was recorded daily until the end of the experiment (15 dpi).

### Assessment of clinical signs

Respiratory and ocular signs were monitored daily during the observation period, from day one to day 15 post-infection. The scoring was as follow; score 0 =  normal breathing, normal conjunctiva and no discharge; score 1 = discrete tracheo-bronchial rales, mild dyspnea with open-mouthed breathing, conjunctiva with slight swelling and no eye closure; score 2 = moderate rales, panting with neck extended and moderate sneezing, swollen conjunctiva and/or partial eye closure; score 3 =  intense rales associated with other symptoms (e.g., intense sneezing, runny nose, cough, sinusitis), severe conjunctivitis, complete eye closure and/or eye discharge. Total clinical signs scores (CSS) per chicken were recorded daily after challenge, and then the median clinical score was calculated for each group.

### Viral shedding

To assess viral shedding, oropharyngeal swabs were collected at 1, 3, 5, 7, 10, and 14 dpi. The swabs were immediately suspended in 1 mL PBS and kept at -20°C until further use. Viral RNA was extracted using the Kylt® RNA/DNA Purification Kit according to the manufacturer’s instructions (AniCon Labor GmbH Laboratory, Germany). The virus titer of each sample was determined using real-time RT-PCR using H9-specific primers and probe sets for conserved regions in the HA2 subunit as described by Monne et al, [30]. The target gene was amplified using Invitrogen™ SuperScript™ III One-Step RT-PCR Kit (Thermo Fisher Scientific, Göteborg, Sweden) on a 7500 Real Time PCR System (Applied Biosystems, Foster City, USA), using the following program: the reverse transcriptase step conditions for primer sets were one cycle at 50°C for 30 min, 95°C for 15 min followed by 45 cycles of 95°C for 15 s, 56°C for 15 s, and 72°C for 15 s. PCR amplifications were performed in 96-well plates in a final volume of 25μl containing 5μl of RNA template, 12.5μl SuperScriptRT-PCR Master Mix, 1 μl of each primer (10μM), 0,5μl TaqMan probe (10μM), 0,5μl of SuperScript™ III Reverse Transcriptase and 4,5 µ L of nuclease free water. To determine virus shedding titer, the cycle threshold (Ct) values of samples were converted to corresponding viral load on Log_10_ as described in previous studies [35]. The excel table illustrating the quantitative estimation of viral concentration from qRT-PCR data using a regression equation can be consulted on Supporting information data (S1 Appendix).

### Gross pathological examination

Macroscopic lesions caused by H9N2 infection were investigated through a series of necropsies on an average of 10 birds per group at 5, 10 and 15 dpi as well as found dead birds from each group. Gross pathological observations were primarily focused on the sinuses, trachea, lungs, and air sacs. Tissue samples from the trachea and lungs were collected for histopathological examination and scored based on previously established methods [11].

### Histopathology examination

Tissue samples from trachea and lungs were fixed in 10% Neutral Buffered Formalin (NBF) for 24-48h. Coronal and longitudinal sections from the samples were dehydrated by immersion in a series of alcohol baths of increasing strength (from 70% to 100%) and embedded in paraffin by a routine procedure. Dewaxed 3-5μm thick sections were stained with hematoxylin and eosin (H&E) [36] and examined using a light microscope. Microscopic lesions were evaluated based on a mean of total individual microscopic lesion scores (MILS) noted in lung and trachea of birds from experimental groups as previously described by [37,38].

### Statistical analysis

Statistical analysis was conducted using IBM SPSS Statistics (version 28.0.1.1). A one-way analysis of variance (ANOVA) followed by a Duncan post-hoc-test was used to analyze the data. The severity of clinical signs, as well as gross and histopathological lesions, were classified using a scoring system from 0 to 3, referring to “normal”, “mild”, “moderate” and “severe” for most observed parameters. For statistical evaluation, the sum of scores and percentage of each evaluated parameter were calculated. The number of birds shedding virus was tested for statistical significance using Fisher’s exact test, and a two-way ANOVA was used to evaluate virus titers in swabs. P-values less than 0.05 were considered significant (P < 0.05).

## Results

### Body weight gain

**Fig 2:**
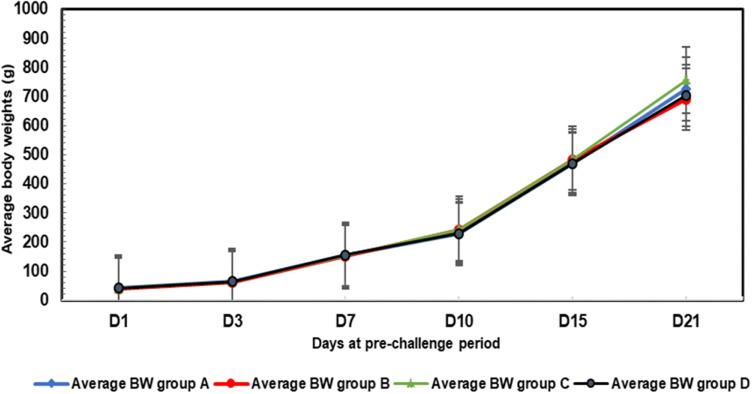
Average body weight trends for the four groups during the pre-challenge period.

During the pre-challenge period, between day 1 and day 21 of age, the average body weights of the four groups (A, B, C and D) followed the same pattern and remained close to one another (**Fig 2**). During this period, no significant difference was observed between the groups, as the p-value for the intergroup sum of squares reached 0.152 ( > 0.05).

**Fig 3:**
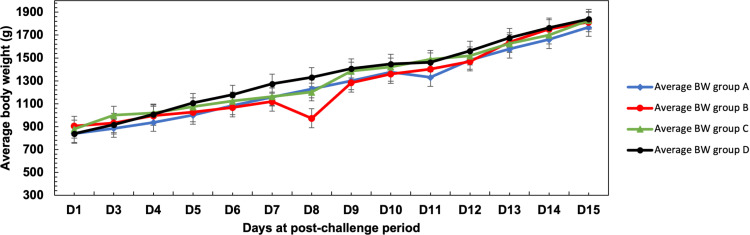
Average body weight trends for the four groups during the post-challenge period.

During the post-infection period, Group A (Challenged with LPAI H9N2 strain isolated in 2016) showed a steady evolution of mean body weights without a specific drop. In contrast, Group B (challenged with the H9N2 strain isolated in 2022) showed a sharp decline in weight between days 7 and 8 post-infection, followed by a recovery that brought their weight close to the levels observed in Group A by day 9 (Fig 3). The significant weight loss in group B correlated with the highest respiratory and ocular symptom scores, which may be explained by the worsening of respiratory symptoms, dehydration and decrease of feed consumption. Group C (not challenged and only subjected to cold stress) showed no drop of body weight, indicating that the low temperature (14°C for 6 hours) alone does not affect production performance (body weight). On the other hand, the average body weights of chicks from the negative control group (D) followed a steady progression, with relatively higher values at the end of the challenge, although they remained close to the values seen in the other groups (Fig 3).

However, the F-test, which compares intergroup variance with intragroup variance, gives a score of 0.266. This value is accompanied by a p-value of 0.850, indicating that there is no significant difference between the groups in terms of body weight gain. Despite this, we noted a drop of weight in Group B which coincides with the highest scores of respiratory symptom scores. Overall, there was no significant difference among the groups, despite the drop-in weight noted in group B at 8 dpi (S2 Appendix).

### Evolution of respiratory symptom scores

The evolution of mean respiratory symptom scores during the post-challenge period showed that groups A and B reached a maximum means of 2.36 and 2.70 on 6 dpi and 5 dpi, respectively. Group C, which received only cold stress, exhibited regressing mean respiratory symptom scores, with a maximum value of 1.70 noted on 2 dpi, decreasing to zero by 7dpi. This suggests that cold stress factor alone may generate moderate respiratory symptoms, but they tend to regress and disappear shortly after exposure. Birds of group D (control group) did not show any respiratory symptoms throughout the post-challenge period. No significant difference was noted between groups C and D for respiratory symptom scores (P = 0.44).

**Fig 4:**
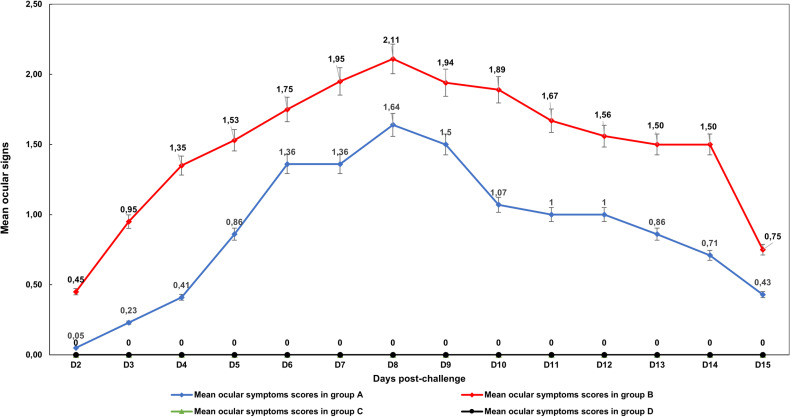
Trends of average respiratory symptom scores during the post-challenge period.

These results illustrated that the challenge model effectively reproduced the respiratory symptoms characteristic of infection with LPAI virus subtype H9N2. The combination of cold-stress and virus inoculation induced a detectable and measurable clinical form of infection. Therefore, this model of challenge can be used as an alternative to co-infections with other pathogens. Support of these findings is presented in **Fig 4** and S3 Appendix.

### Evolution of ocular symptom scores

**Fig 5:**
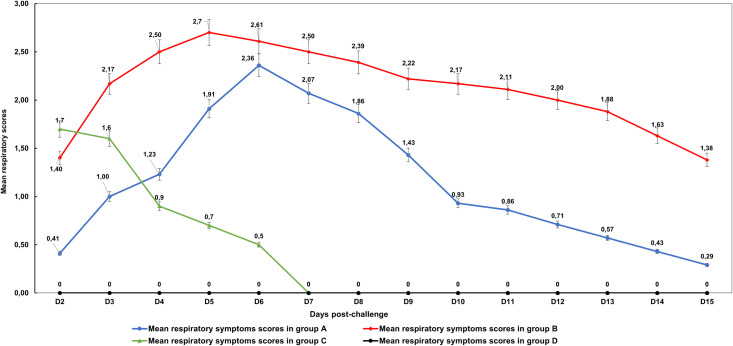
Trends of average ocular symptom scores during the post-challenge period.

The curves showing the evolution of average ocular symptom scores during the post-challenge period showed that the maximum score values for groups A and B were recorded on 8 dpi, with mean scores of 1.61 and 2.11 respectively (Fig 5).

No statistical difference was noted between groups C and D, as indicated by the same superscript letters presented in S3 Appendix.

### Mortality rate

No mortality was observed in groups A, C, and D. Mortality was only noted in group B (infected with H9N2 virus isolated in 2022), with a rate of 10% (3/30). Statistical analysis revealed a significant difference between group B and other groups (P = 0.019).

### Gross pathology post-challenge lesions

The three cases of mortality in group B were found on 6 and 13 dpi. The two birds died on 6 dpi exhibited airsaculitis, congestive tracheitis, fibrinous plugs, fibrinous pneumonia and sero-fibrinous sinusitis. The cause of death was probably the obstruction of the bronchial lumen by fibrin casts at the tracheal bifurcation.

**Fig 6:**
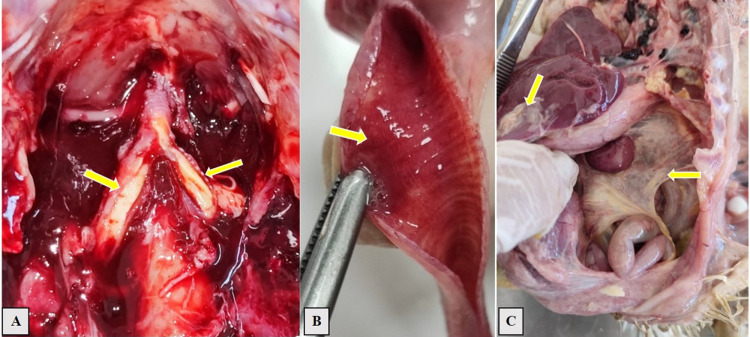
Necropsy lesions of deceased chicks from group B, lesions are presented in yellow arrows. A) Fibrinous plug in the tracheal bifurcation observed at 6 dpi. B) Congested trachea and hyperemia noted at 6 dpi. C) Fibrinous airsacculitis and fibrinous perihepatitis observed at 13 dpi.

The bird that died on 13^th^ dpi showed fibrinous airsacculitis, fibrinous perihepatitis, and fibrinous pneumonia (Fig 6).

From the series of necropsies held at 5 dpi, birds of group C showed slight mucous and a mild redness congestion of trachea. In contrast, birds of group A and B showed 100% mild to severe congestive tracheitis. Group A had 50% of birds with fibrinous tracheitis, and group B had 60%. Fibrinous plugs at the tracheal bifurcation were observed in 10% of birds of group A and 40% of group B. Pneumonia was noted in 20% of individuals from group A and 100% from group B, while fibrinous pneumonia was noted at 10% of sacrificed birds from group B.

Fibrinous sinusitis observed at 5dpc was at rates of 20% and 10% in groups A and B, respectively. Statically, significant differences were noted between groups A and B (P < 0.05), except lesions of fibrinous airsaculitis, which were noted in 20% of flocks in both groups (P = 0.251). These results are detailed in S4 Appendix.

At 10 dpi, the severity of congestive tracheitis lesions had decreased to 70% and 80% in scarified birds from groups A and B, respectively, with a statistically significant difference (P = 0.001). Fibrinous tracheitis was observed at rate of 10% in both groups, with no significant difference (P = 0.45). Group A displayed fewer gross pathological lesions, with 20% of birds exhibiting congestive pneumonia and 10% showing fibrinous airsaculitis and sinusitis. In contrast, birds from Group B had higher rates of lesions: 60% showing congestive pneumonia, 10% with fibrinous pneumonia, 40% with fibrinous airsaculitis, and 20% with fibrinous sinusitis. Notably, no group showed fibrinous plugs at 10 dpi. These differences between the groups were statistically significant, as detailed in S4 Appendix.

At 15 dpi, lesions had regressed in autopsied birds from group A; mild congestive tracheitis and congestive pneumonia were noted in 40% of chicks, serous sinusitis in 30%, and catarrhal tracheitis and catarrhal sinusitis in 10%. No individuals presented with fibrinous tracheitis, fibrinous plugs, fibrinous pneumonia, fibrinous airsaculitis, or fibrinous congestive sinusitis. Conversely, Group B showed significantly higher rates of lesions: 88% had congestive pneumonia (P = 0.001), 63% had congestive tracheitis (P = 0.002), and 38% had congestive sinusitis and catarrhal sinusitis (*P = 0.091* and *P = 0.287*). No birds in Group B showed fibrinous tracheitis, fibrinous plugs, fibrinous pneumonia, fibrinous airsaculitis, or fibrinous sinusitis (S4 Appendix).

### Histopathological lesions

Lungs and trachea of challenged chicks were subjected to microscopical investigations. In trachea, hyperplasia, loss of cilia and the presence of exudate on the epithelial surface and lumen were found, over the three necropsy days, in 60% of birds from group A and 80% from group B. The mean individual lesion score (MILS) was higher in group B (1.85) compared to group A (1.25) but with no statistical difference noted (*P = 0.41*). Additionally, changes in lamina propria were also noted in 90% of individuals from group B, with a MILS of 1.75 and in 70% of individuals from group A, with a MILS of 1.30 without statistical difference (*P = 0.63*) between the two groups. Individuals from group C, having received only cold stress, showed very few mild lesions with lower MILS, not exceeding 2.. These changes were associated with pneumonia extending to only 2% of the lung fragment surface. Mild loss of cilia was noted in trachea of 10% of examined chicks from this group. Despite the difference in total tracheal scores, no statistical difference was shown (*P* = 0.53) as detailed in Table 2.

**Table 2:** Average histopathological lesions and scores noted in trachea and lungs of birds during post-infection period.

Organ	Microscopic changes and mean scores	Groups				P-value
**A**	**B**	**C**	**D**
**Lung**	**Fibrinous suppurative bronchitis**	40%^a^ (6.4)	90%^b^ (13.44)	0%^c^	0%^c^	0.002
	**Changes in interstitial inter-lobular spaces**	50%^a^ (1.00)	90%^b^ (2.64)	50%^a^ (1.00)	0%^c^	0.004
	**Fibrinous suppurative pneumonia**	60%^a^ (2.7)	90%^a^ (3.33)	0%^c^	0%^c^	0.21
	**Extent of pneumonia**	6%	11%	2%	0%	
	**Total pulmonary scores**	**10.1** ^a^	**21.41** ^b^	**1** ^c^	**0** ^c^	0.002
**Trachea**	**Epithelial changes**	60%^a^ (1.25)	80%^a^ (1.85)	10%^b^ (1.00)	0%^c^	0.41
	**Lamina Propria changes**	70%^a^ (1.3)	90%^a^ (1.75)	0%^c^	0%^c^	0.63
	**Total trachea scores**	**2.55** ^a^	**3.6** ^a^	**1** ^a^	**0** ^a^	0.53

Different superscript letters in the same row indicate a significant difference (P < 0.05).

**Fig 7:**
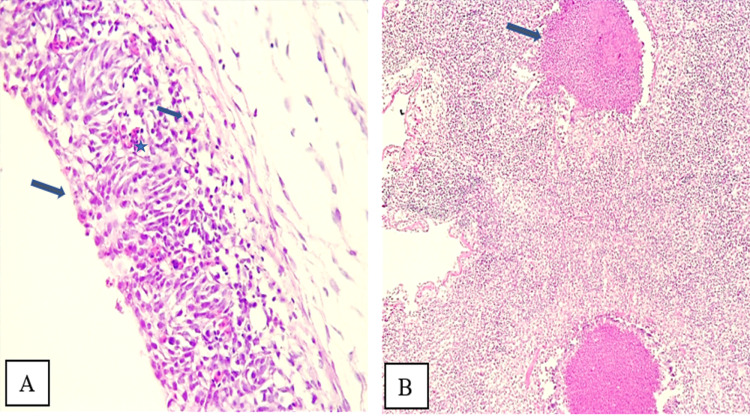
Microscopical lesions in trachea and lungs H&E staining on dpi 5. A) Moderate tracheitis with epithelial hyperplasia (Asterisk), loss of cilia and inflammatory cellular infiltrate of lamina propria (Arrows) in a chick from group A. B) Fibrino-suppurative pneumonia with locally diffuse inflammatory cell infiltrate and a large plug of inflammatory exudate within a parabronchi (Arrow) a chick from group B. (H&E, X10).

**Fig 8:**
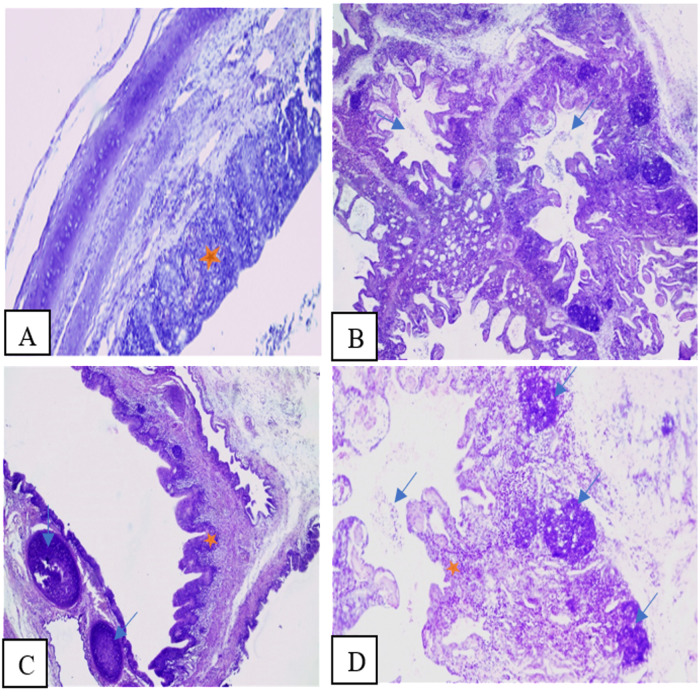
Microscopical lesions in trachea and lungs H&E staining on dpi 10. A) Moderate to severe subacute tracheitis (score 2) with epithelial hyperplasia (Asterisk) and mixed inflammatory cell infiltrate of the lamina propria in an individual from group A. B) Moderate pneumonia with inflammatory exudate in the lumens of the atria and parabronchi (Arrows) noted in a chick from group A. C) Mild to moderate chronic bronchitis (score 2) with epithelial hyperplasia (Asterisk) and mononuclear cell infiltrate of the lamina propria and prominent nodular development of bronchoalveolar lymphoid tissue (BALT) (Arrow) noted in a chick in group B. D) Moderate chronic bronchitis with mild exudate in the bronchial lumen (Arrow) and mononuclear cell infiltrate of the lamina propria and surrounding interstitial tissue (Asterisk) and prominent nodular BALT (Arrows) development noted in a bird of group A. (H&E, X10).

In the lungs, fibrinous-suppurative bronchitis associated to hyperplasia and loss of cilia on primary and secondary bronchi were found in 40% of birds from groups A and 90% from group B. Furthermore, 50% and 90% of samples from groups A and B, respectively, exhibited changes in the interstitial inter-lobular spaces, characterized mainly by inflammatory cellular infiltration, edema and hyperemia. The MILS were 1,00 in group A and 2,64 in group B, showing a significant difference (P = 0.004). Diffuse fibrinious suppurative pneumonia was observed in 60% of samples from group A and 90% of group B, with mean individual scores of 1,89 and 2,64, respectively, presenting no statistical difference (P = 0.21). The extent of pneumonia was higher in group B (11%) compared to group A (6%).. Those findings were illustrated in **Figs 7** and 8 presenting changes noted in 5 and 10 dpi in both challenged groups.

### Quantification of viral shedding

The viral shedding titers of LPAI H9N2 during the post infection period was monitored at 3, 6, 9, 11, 13 and 15 dpi. Oropharyngeal shedding in group B had significantly higher mean viral load at 3 dpi (4.62 log₁₀) compared to group A (3.57 log₁₀) (*P = 0.021*).

**Fig 9:**
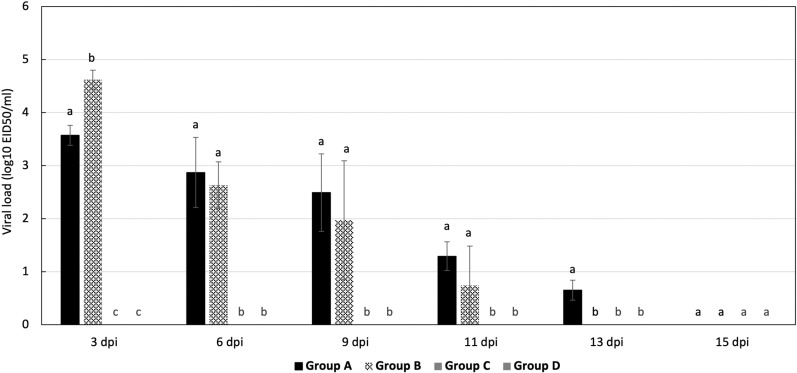
Mean AIV-H9N2 shedding titer (Log_10_) in oropharyngeal swabs. Data are presented as means ±  SE. Bars with no common superscript were significantly different at P ≤ 0.05.

At 6, 9, 11, and 13 dpi, the mean viral loads were higher in group A; 2.87 log₁₀, 2.49 log₁₀, 1.29 log₁₀ and 0.65 log₁₀ respectively, although no statistically significant difference was noted compared to group B (P = 0.54). Additionally, the duration of oropharyngeal viral shedding was shorter in group B compared to group A, while in group B it continued up to 13 dpi (Fig 9). Although, there was no viral shedding in group C, which receive only stress cold, and the negative control (group D).

## Discussion

Avian influenza is one of the most significant threats for the poultry industry worldwide. The H9N2 subtype is highly widespread in turkey and chicken populations across many regions, especially in Africa and Asia. Countries like China, South Korea, Pakistan, Iran, UAE, Morocco and Egypt have implemented systematic vaccination strategies as a crucial measure to control and prevent the spread of the virus in poultry [8,39–43]. In Morocco, the endemic circulation of avian influenza has resulted in substantial economic losses, marked by high mortality rates and significant decrease in production performances. This situation has raised concerns about the virulence of the virus that may have increased since its initial introduction in 2016. Several genetic studies suggest that the viral evolution of H9N2 in Morocco may have implications for its host range and potential virulence. H9N2 strains circulating since 2021 has exhibited notable nucleotide and amino acid variations, ranging from 1-6% compared to the 2016 strain. These differences include mutations at various genomic sites and changes in glycosylation patterns, which potentially influence viral characteristics [9]. Furthermore, evolutionary and network analyses between different temporal groups of H9N2 viruses in Morocco showed clear genetic divergence. The 2016-2017 HA sequences showed high similarity within their group, but substantial genetic distance separates them from the 2018-2019 and 2020-2021 strains [44].

Additionally, several factors also influence the virulence such as management practices, co-infections with bacterial, viral, or fungal, immunosuppressive agents, and the age and breed of chickens [45–48]. The discrepancy between pathogenicity observed in experimental and field conditions has been widely studied. In this context, to better evaluate the notable severity of H9N2 under experimental condition, a challenge model using cold stress was developed to reproduce the clinical symptoms observed in field without co-infections with other pathogens.

Cold stress protocol was implemented at 14°C, as temperatures below 18°C are known to induce cold-related stress responses, tissue damage, and potential mortality in poultry. This specific temperature selection was based on previous research demonstrating significant impacts on chicken health and performance [49–52]. Studies by Mendes et al. (1997) [50] showed that exposure to 15.5°C negatively affected growing chickens’ feed intake, feed conversion ratio, body weight, and mortality rates, while also leading to conditions such as ascites and cardiomyopathy. Additionally, Zhang et al. (2014) [53] demonstrated that both acute (12 ±  1°C) and chronic cold stress conditions induced oxidative stress in broiler duodenum. The timing of our cold stress protocol was carefully designed to stimulate field conditions. Based on the observation by Shinder et al. (2002) [54], birds can adjust to temperature variations within 24 hours, as evidenced by corticosterone levels. This timeframe was carefully selected to induce measurable stress responses while preventing physiological adaptation that could potentially mask H9N2 infection severity [51,55]. To prevent this adaptation and maintain the stress response, we implemented a protocol that mimicked natural diurnal temperature fluctuations and timed the viral challenge to coincide with the cold stress period. This approach, supported by Zhao et al. (2013) [49], was specifically chosen to maximize the impact on host-pathogen interactions while reflecting field conditions.

Experimental infections of commercial broiler chickens with LPAIV H9N2 strains generally lead to moderate signs of illness including depression, crouching, decreased in feed and water consumption and respiratory symptoms such as coughing, sneezing, gasping, eye and/or facial swelling, and nasal discharge [11,22,48]. Despite these symptoms, the experimental conditions often do not replicate the high virulence observed in the field, leading to a moderate impairment of the general condition of the chickens [46,56,57].

Our study revealed that body weight gain (BWG) from (day 1 to day 35) showed no significant differences between the groups. However, Group B, which was challenged with the recently isolated H9N2, experienced a progressive decline in weight from day 5 post-challenge, with a significant drop on the 8^th^ dpi, followed by a gradual recovery. The substantial weight loss in Group B coincided with the peak of respiratory and ocular symptom scores, likely due to the worsening of the disease. Although limited data are available on the impact of H9N2 LPAI on growth performance, Substain et al, (2011) [56] reported that weight loss could be associated with H9 virus infection in birds. Moreover, in group C, a sudden variation in body weight was observed between days 22 and 35. This may be attributed to individual differences among the animals or external environmental factors that specifically affected certain individuals within the group, despite all groups being maintained under the same conditions. Additionally, the sample size in the groups may have been insufficient to adequately account for such variability, potentially leading to an inflated standard deviation.

Regarding the respiratory and ocular signs observed in our study, the control birds exhibited no obvious clinical signs or mortality. In contrast, birds in Group B exhibited significantly higher and earlier peak in mean respiratory symptom scores, reaching 2.7 at 5 dpi, compared to 2.36 at 6 dpi in Group A. This peak occurred earlier than what was reported by Khantour et al. (2021) [58], who observed that clinical signs in groups challenged with the first isolated H9N2 strain in Morocco (A/ chicken/Morocco/01/2016), appeared from 2 dpi, persisted until 12 dpi and peaked at 9 dpi. Other studies have similarly reported that H9N2 infection symptoms appeared at 2–3 dpi, lasted until 5–7 dpi, and usually resolved by 10–12 dpi without mortality [56,59]. The observed differences in the timing and severity of clinical signs in our study may be attributed to several factors. First, prolonged viral replication in tissues and the increased pathogenicity of the 2022 strain compared to the 2016 strain could explain the earlier onset and higher peak of respiratory symptoms. Second, the stress experienced by our chickens due to experimental conditions—such as handling, environmental changes, or the challenge protocol itself—may have contributed to the exacerbation and earlier manifestation of clinical signs.

For assessment of ocular sings, the peak in both challenged groups were reached at 8 dpi and progressively regressed for the rest of the challenge period, lasting until 15 dpi. Evaluating conjunctivitis as a clinical proxy was supported by Kim et al (2013) [60] for assessing AIV vaccine efficacy. His study presented a significant difference between two SPF chicks groups vaccinated and unvaccinated against H9N2, challenged by bi-ocular instillation with the virus. In contract to our findings, Spackman et al, (2011) [35], reported only mild, transient conjunctivitis lasting from 3 to 5 days after ocular challenge with LPAI H7N2 in broilers, SPF Leghorns and turkeys. Spackman and co-authors also noted a more severe impact on the challenged eye than on the opposite eye.

The mortality rate of 10% (3/30) observed in Group B, compared to no mortalities in the other groups, is notably higher than previously reported in experimental studies. Previous research has shown that experimental inoculation with H9N2 alone typically results in no or low mortality rates, despite the virus replicating and causing localized lesions in the respiratory tract [46,56,57]. In some cases, mortality rates in experimental settings have been reported to be around 3.3% [61], 5% [11], and generally do not exceed 6% [62]. In contrast, mortality rates in naturally infected farms are typically much higher, ranging from 5% to 30% depending on factors such as co-infections, vaccination status, and farm management practices [11,14,63]. This variability under field conditions is often exacerbated by concurrent bacterial infections, which play a significant role in disease progression, particularly for low to moderately pathogenic influenza strains [64]. Among the contributing factors, cold stress has been shown to play a crucial role in exacerbating H9N2 infection outcomes. It can compromise the immune system, increase susceptibility to viral infections, and predispose birds to secondary bacterial complications. Additionally, it may reduce feed intake and metabolic efficiency, further exacerbating weight loss and increasing mortality [52,53,65]. Future studies can be conducted to examine the effects of cold stress applied either before or after AI challenge to better mimic field conditions, such as seasonal temperature fluctuations. This would provide valuable insights into how environmental stressors interact with H9N2 pathogenesis and help to further understand the full scope of the virus’s impact under varying environmental conditions.

The distinct pathological profile of 2022 H9N2 strain was clear during the necropsy examinations. We noted a higher incidence of congestive and fibrinous tracheitis, fibrinous plugs and fibrinous pneumonia at 5 dpi in group B. This increased severity and frequency of lesions in this group is in line with what was reported by Bona et al, (2023) [66] when comparing A/chicken/Morocco/2021 H9N2 virus with Egyptian and Saudi Arabian strains. Furthermore, during our macroscopic examination, the most significant lesions were in respiratory airways specifically the sinuses, trachea, bronchioles and to a lesser extent, the air sacs and lungs and tended generally to recover by 10 and 15dpi. These findings align with previous reports by Bano et al (2003), Gharaibeh (2008), Swayne and Pantin-Jackwood (2008), Awadin et al, (2018) and El khantour et al (2021) [46,58,63,67,68],

The macroscopic lesion pattern observed in group B, challenged with 2022 H9N2 strain, corresponded closely with the microscopic findings, indicating a pronounced pathogenic impact. This group exhibited significantly higher rates of severe changes in trachea and lungs compared to group A, challenged with the 2016 strain. Notably, the extent of pneumonia was greater in group B, affecting 11% of the lung surface compared to only 6% in Group A, suggesting a more aggressive viral replication and tissue damage. Previous studies confirmed that histopathological changes were predominant in the respiratory tract, characterized by inflammatory with exfoliating epithelial cells, epithelial degeneration and necrotic processes. The proliferation of goblet cells was first seen in the trachea, followed by sloughing of the tracheal epithelium, deciliation and lymphocytic infiltration of the mucosa. Lesions in the lungs were characterized by congestion, interstitial pneumonia, bronchitis, catarrhal infiltration, edema around the blood vessels with heterophil cell infiltration, and collapse of alveoli. Pale bluish mucus and heterophil accumulation in the lumen of secondary bronchioles were also present [56,66,67,69].

The monitoring of viral shedding using oropharyngeal swabs tested by RT-PCR revealed that group B excreted higher viral loads (4.62 log₁₀ vs. 3.57 log₁₀ in Group A at 3 dpi) over a shorter duration, ceasing at 11 dpi compared to 13 dpi in Group A. These findings suggest that genetic changes in the 2022 H9N2 strain have influenced its replication dynamics and shedding patterns, leading to an earlier peak compared to previous study where peak shedding occurred between 5 and 7 dpi in unvaccinated birds [70]. However, these results are consistent with Bortolami et al. (2022) [71] findings, who showed that, for the unvaccinated challenged group with H9N2 strain (A/Chicken/Saudi Arabia/3622-31/13) at a dose of 10^6^ EID50, the peak was occurred between 2 and 3 dpi.

Therefore, the combination of cold-stress and viral challenge allows for the clinical form of the infection to be detectable and measurable using the established “scoring” system, making it a suitable challenge model instead of H9 co-infections with other pathogens. A/chicken/Morocco/178-2/2022(H9N2) proved to be more virulent by several criteria used in our study compared to a strain firstly introduced in Morocco in 2016. However, further investigations may help to identify the specific factors contributing to the differences in virulence between the H9N2 strains.

## Conclusion

From this study, we conclude that the experimental model using cold as a stress factor to exacerbate clinical and necropsy signs of LPAI virus subtype H9N2 in conventional broilers is valid. This model successfully used to mimic the field conditions, providing a reliable basis for assessing virulence and pathogenicity. Our research revealed a significant difference in pathogenicity between strains isolated in 2016 and 2022: A/chicken/Morocco/SF1/2016 (H9N2) and A/chicken/Morocco/178-2/2022 (H9N2), both belonging to the G1 lineage. The 2022 H9N2 strain demonstrated a significantly higher pathogenicity compared to the 2016 strain. These findings underscore the importance of ongoing surveillance and research to understand the evolving nature of H9N2 and its impact on poultry health. Further studies should focus on identifying the genetic determinants responsible for this increase in virulence. Such research will be crucial for developing effective strategies and compatible vaccination programs for managing and controlling LPAI H9N2 infections in poultry.

## Supporting information

S1 Appendix Quantitative estimation of viral concentration from qRT-PCR data using a regression equation. (XLSX)

S2 Appendix Body weight gains during the different periods of the experiment. (PDF)

S3 Appendix Clinical mean respiratory and ocular symptom scores. (PDF)

S4 Appendix Gross pathological lesions observed on the respiratory tract at 5, 10 and 15 dpi. (PDF)

## Abbreviations

LPAIV: low pathogenic avian influenza virus
CESASPV: Ethical Committee for Animal Veterinary Science and Public Health
WOAH: World Organization for Animal Health; EID, embryo infectious dose
HI: hemagglutination inhibition
HAU: hemagglutinin units;
DPI: , day post-infection
BWG: body weight gain
CSS: clinical signs scores
MILS: Mean individual lesion score.

## References

[1] European Food Safety Authority, European Centre for Disease Prevention and Control, European Union Reference Laboratory for Avian Influenza, Adlhoch C, Fusaro A, Gonzales JL, et al. Avian influenza overview December 2022 - March 2023. EFSA J. 2023;21(3):e07917. doi: 10.2903/j.efsa.2023.7917 36949860 PMC10025949

[2] Homme PJ, Easterday BC. Avian influenza virus infections. I. Characteristics of influenza A-turkey-Wisconsin-1966 virus. Avian Dis. 1970;14(1):66–74. doi: 10.2307/1588557 4314007

[3] Pusch EA, Suarez DL. The multifaceted zoonotic risk of H9N2 Avian influenza. Vet Sci. 2018;5(4):82. doi: 10.3390/vetsci5040082 30248906 PMC6313933

[4] Song W, Qin K. Human-infecting influenza A (H9N2) virus: a forgotten potential pandemic strain?. Zoonoses Public Health. 2020;67(3):203–12. doi: 10.1111/zph.12685 31930694

[5] Wang H, Zhang Z, Chen Z, Zhang Y, Lv Q, An X, et al. High genetic diversity and frequent genetic reassortment of avian influenza A(H9N2) viruses along the East Asian-Australian migratory flyway. Infect Genet Evol. 2016;39:325–9. doi: 10.1016/j.meegid.2016.02.013 26876220

[6] Guan Y, Shortridge KF, Krauss S, Webster RG. Molecular characterization of H9N2 influenza viruses: were they the donors of the “internal” genes of H5N1 viruses in Hong Kong? Proc Natl Acad Sci U S A. 1999;96(16):9363–7. doi: 10.1073/pnas.96.16.9363 10430948 PMC17788

[7] Nagy A, Mettenleiter TC, Abdelwhab EM. A brief summary of the epidemiology and genetic relatedness of avian influenza H9N2 virus in birds and mammals in the Middle East and North Africa. Epidemiol Infect. 2017;145(16):3320–33. doi: 10.1017/S0950268817002576 29168447 PMC9148743

[8] El Houadfi M, Fellahi S, Nassik S, Guérin J-L, Ducatez MF. First outbreaks and phylogenetic analyses of avian influenza H9N2 viruses isolated from poultry flocks in Morocco. Virol J. 2016;13(1):140. doi: 10.1186/s12985-016-0596-1 27527708 PMC4986173

[9] Arbani O, Ducatez MF, Mahmoudi S, Salamat F, Khayi S, Mouahid M, et al. Low pathogenic avian influenza H9N2 viruses in Morocco: antigenic and molecular evolution from 2021 to 2023. Viruses. 2023;15(12):2355. doi: 10.3390/v15122355 38140596 PMC10747644

[10] Bóna M, Földi J, Dénes L, Harnos A, Paszerbovics B, Mándoki M. Evaluation of the virulence of low pathogenic H9N2 avian influenza virus strains in broiler chickens. Vet Sci. 2023;10(12):671. doi: 10.3390/vetsci10120671 38133222 PMC10747561

[11] Nili H, Asasi K. Natural cases and an experimental study of H9N2 avian influenza in commercial broiler chickens of Iran. Avian Pathol. 2002;31(3):247–52. doi: 10.1080/03079450220136567 12396348

[12] Umar S, Guerin JL, Ducatez MF. Low Pathogenic avian influenza and coinfecting pathogens: a review of experimental infections in avian models. Avian Dis. 2017;61(1):3–15. doi: 10.1637/11514-101316-Review 28301244

[13] Liu H, Chen Y, Li H, Yang L, Yang S, Luo X, et al. Pathogenicity, transmissibility, and immunogenicity of recombinant H9N2 avian influenza viruses based on representative viruses of Southeast China. Poult Sci. 2023;102(6):102625. doi: 10.1016/j.psj.2023.102625 37004288 PMC10090987

[14] Peacock THP, James J, Sealy JE, Iqbal M. A global perspective on H9N2 avian influenza virus. Viruses. 2019;11(7):620. doi: 10.3390/v11070620 31284485 PMC6669617

[15] Belkasmi SFZ, Fellahi S, Touzani CD, Faraji FZ, Maaroufi I, Delverdier M, et al. Co-infections of chickens with avian influenza virus H9N2 and Moroccan Italy 02 infectious bronchitis virus: effect on pathogenesis and protection conferred by different vaccination programmes. Avian Pathol. 2020;49(1):21–8. doi: 10.1080/03079457.2019.1656328 31412705

[16] Ellakany HF, Gado AR, Elbestawy AR, Abd El-Hamid HS, Hafez HM, Abd El-Hack ME, et al. Interaction between avian influenza subtype H9N2 and Newcastle disease virus vaccine strain (LaSota) in chickens. BMC Vet Res. 2018;14(1):358. doi: 10.1186/s12917-018-1689-4 30458777 PMC6245631

[17] Sid H, Benachour K, Rautenschlein S. Co-infection with multiple respiratory pathogens contributes to increased mortality rates in algerian poultry flocks. Avian Dis. 2015;59(3):440–6. doi: 10.1637/11063-031615-Case.1 26478165

[18] Arafat N, Abd El Rahman S, Naguib D, El-Shafei RA, Abdo W, Eladl AH. Co-infection of Salmonella enteritidis with H9N2 avian influenza virus in chickens. Avian Pathol. 2020;49(5):496–506. doi: 10.1080/03079457.2020.1778162 32835500

[19] Kishida N, Sakoda Y, Eto M, Sunaga Y, Kida H. Co-infection of *Staphylococcus aureus* or *Haemophilus paragallinarum* exacerbates H9N2 influenza A virus infection in chickens. Arch Virol. 2004;149(11):2095–104. doi: 10.1007/s00705-004-0372-1 15503199

[20] Mosleh N, Dadras H, Asasi K, Taebipour MJ, Tohidifar SS, Farjanikish G. Evaluation of the timing of the *Escherichia coli* co-infection on pathogenecity of H9N2 avian influenza virus in broiler chickens. Iran J Vet Res. 2017;18(2):86–91. 28775746 PMC5534249

[21] Pan Q, Liu A, Zhang F, Ling Y, Ou C, Hou N, et al. Co-infection of broilers with *Ornithobacterium rhinotracheale* and H9N2 avian influenza virus. BMC Vet Res. 2012;8 104. doi: 10.1186/1746-6148-8-104 22748160 PMC3424113

[22] Swayne DE. Diseases of poultry. 14th edition. Hoboken, NJ: Wiley-Blackwell; 2020.

[23] Manual of Diagnostic Tests and Vaccines for Terrestrial Animals- Avian Influenza. In OIE Terrestrial Manual 2015. OIE: Rome, Italy, 2015.

[24] Olanrewaju HA, Thaxton JP, Iii WAD, Purswell J, Roush WB, Branton SL. A review of lighting programs for broiler production. Int J of Poul Sci. 2006;5(4):301–8. doi: 10.3923/ijps.2006.301.308

[25] Gratta F, Bošković Cabrol M, Xiccato G, Birolo M, Bordignon F, Trocino A. Effect of light restriction on productive results and behavior of broiler chickens. Poult Sci. 2023;102(12):103084. doi: 10.1016/j.psj.2023.103084 37826901 PMC10568561

[26] Reed LJ, Muench H. A simple method of estimating fifty per cent endpoints12. Am J Epidemiol. 1938;27(3):493–7. doi: 10.1093/oxfordjournals.aje.a118408

[27] Hu M, Jin Y, Zhou J, Huang Z, Li B, Zhou W, et al. Genetic characteristic and global transmission of influenza A H9N2 virus. Front Microbiol. 2017;8:2611. doi: 10.3389/fmicb.2017.02611 29312274 PMC5744263

[28] Yang Q, Ji J, Yang J, Zhang Y, Yin H, Dai H, et al. Diversity of genotypes and pathogenicity of H9N2 avian influenza virus derived from wild bird and domestic poultry. Front Microbiol. 2024;15:1402235. doi: 10.3389/fmicb.2024.1402235 38974026 PMC11225357

[29] Alexander DJ. Summary of avian influenza activity in Europe, Asia, Africa, and Australasia, 2002-2006. Avian Dis. 2007;51(1 Suppl):161–6. doi: 10.1637/7602-041306R.1 17494548

[30] Monne I, Ormelli S, Salviato A, De Battisti C, Bettini F, Salomoni A, et al. Development and validation of a one-step real-time PCR assay for simultaneous detection of subtype H5, H7, and H9 avian influenza viruses. J Clin Microbiol. 2008;46(5):1769–73. doi: 10.1128/JCM.02204-07 18367569 PMC2395090

[31] Lovegrove BG. Modification and miniaturization of Thermochron iButtons for surgical implantation into small animals. J Comp Physiol B. 2009;179(4):451–8. doi: 10.1007/s00360-008-0329-x 19115060

[32] El Allali K, Achaâban MR, Bothorel B, Piro M, Bouâouda H, El Allouchi M, et al. Entrainment of the circadian clock by daily ambient temperature cycles in the camel (*Camelus dromedarius*). Am J Physiol Regul Integr Comp Physiol. 2013;304(11):R1044–52. doi: 10.1152/ajpregu.00466.2012 23485867

[33] Farsi H, Harti D, Achaâban MR, Piro M, Raverot V, Bothorel B, et al. Melatonin rhythm and other outputs of the master circadian clock in the desert goat (*Capra hircus*) are entrained by daily cycles of ambient temperature. J Pineal Res. 2020;68(3):e12634. doi: 10.1111/jpi.12634 32011000

[34] Harris RBS. Consuming sucrose solution promotes leptin resistance and site specifically modifies hypothalamic leptin signaling in rats. Am J Physiol Regul Integr Comp Physiol. 2021;320(2):R182–94. doi: 10.1152/ajpregu.00238.2020 33206557 PMC7948127

[35] Spackman E, Senne DA, Myers TJ, Bulaga LL, Garber LP, Perdue ML, et al. Development of a real-time reverse transcriptase PCR assay for type A influenza virus and the avian H5 and H7 hemagglutinin subtypes. J Clin Microbiol. 2002;40(9):3256–60. doi: 10.1128/JCM.40.9.3256-3260.2002 12202562 PMC130722

[36] Bancroft JD. Theory and Practice of Histological Techniques. Elsevier Health Sciences; 2008.

[37] Belkasmi SFZ, Fellahi S, Umar S, Delpont M, Delverdier M, Lucas M-N, et al. Efficacy of massachusetts and 793B vaccines against infectious bronchitis Moroccan-Italy 02 virus in specific-pathogen-free chickens and commercial broilers. Avian Dis. 2017;61(4):466–71. doi: 10.1637/11686-060817-Reg.1 29337615

[38] Nakamura K, Cook JK, Otsuki K, Huggins MB, Frazier JA. Comparative study of respiratory lesions in two chicken lines of different susceptibility infected with infectious bronchitis virus: histology, ultrastructure and immunohistochemistry. Avian Pathol. 1991;20(2):241–57. doi: 10.1080/03079459108418761 18680019

[39] Adel A, Mosaad Z, Shalaby AG, Selim K, Samy M, Abdelmagid MA, et al. Molecular evolution of the hemagglutinin gene and epidemiological insight into low-pathogenic avian influenza H9N2 viruses in Egypt. Res Vet Sci. 2021;136:540–9. doi: 10.1016/j.rvsc.2021.04.006 33887563

[40] Ali M, Khan M-R, Aslam A, ur-Rehman H, Masood S, Masood A, et al. Comparative molecular characterization and pathogenicity of H9N2 avian influenza virus in commercial poultry flocks in Pakistan. Pak Vet J. 2021;41:451–5. doi: 10.2185/jrm.2021-047 35432636 PMC8984621

[41] Bashashati M, Chung DH, Fallah Mehrabadi MH, Lee D-H. Evolution of H9N2 avian influenza viruses in Iran, 2017-2019. Transbound Emerg Dis. 2021;68(6):3405–14. doi: 10.1111/tbed.13944 33259145

[42] Dong J, Zhou Y, Pu J, Liu L. Status and challenges for vaccination against avian H9N2 influenza virus in China. Life (Basel). 2022;12(9):1326. doi: 10.3390/life12091326 36143363 PMC9505450

[43] Sagong M, Lee K-N, Lee E-K, Kang H, Choi YK, Lee Y-J. Current situation and control strategies of H9N2 avian influenza in South Korea. J Vet Sci. 2023;24(1):e5. doi: 10.4142/jvs.22216 36560837 PMC9899936

[44] El Mellouli F, Mouahid M, Fusaro A, Zecchin B, Zekhnini H, El Khantour A, et al. Spatiotemporal dynamics, evolutionary history and zoonotic potential of Moroccan H9N2 Avian influenza viruses from 2016 to 2021. Viruses. 2022;14(3):509. doi: 10.3390/v14030509 35336916 PMC8951762

[45] Aamir UB, Wernery U, Ilyushina N, Webster RG. Characterization of avian H9N2 influenza viruses from United Arab Emirates 2000 to 2003. Virology. 2007;361(1):45–55. doi: 10.1016/j.virol.2006.10.037 17157891 PMC2735206

[46] Bano S, Naeem K, Malik SA. Evaluation of pathogenic potential of avian influenza virus serotype H9N2 in chickens. Avian Dis. 2003;47(3 Suppl):817–22. doi: 10.1637/0005-2086-47.s3.817 14575070

[47] Capua I, Alexander DJ. Avian influenza infections in birds--a moving target. Influenza Other Respir Viruses. 2007;1(1):11–8. doi: 10.1111/j.1750-2659.2006.00004.x 19459279 PMC4634665

[48] Pazani J, Marandi MV, Ashrafihel J, Marjanmehr SH, Ghods F. Pathological studies of A/Chicken/Tehran/ZMT - 173/99 (H9N2) influenza virus in commercial broiler chickens of Iran. Int J Poul Sci. 2008;7(5):502–10. doi: 10.3923/ijps.2008.502.510

[49] Zhao F-Q, Zhang Z-W, Yao H-D, Wang L-L, Liu T, Yu X-Y, et al. Effects of cold stress on mRNA expression of immunoglobulin and cytokine in the small intestine of broilers. Res Vet Sci. 2013;95(1):146–55. doi: 10.1016/j.rvsc.2013.01.021 23419935

[50] Mendes AA, Watkins SE, England JA, Saleh EA, Waldroup AL, Waldroup PW. Influence of dietary lysine levels and arginine:lysine ratios on performance of broilers exposed to heat or cold stress during the period of three to six weeks of age. Poult Sci. 1997;76(3):472–81. doi: 10.1093/ps/76.3.472 9068047

[51] Li D, Tong Q, Shi Z, Zheng W, Wang Y, Li B, et al. Effects of cold stress and ammonia concentration on productive performance and egg quality traits of laying hens. Animals (Basel). 2020;10(12):2252. doi: 10.3390/ani10122252 33266274 PMC7760501

[52] Zhou HJ, Kong LL, Zhu LX, Hu XY, Busye J, Song ZG. Effects of cold stress on growth performance, serum biochemistry, intestinal barrier molecules, and adenosine monophosphate-activated protein kinase in broilers. Animal. 2021;15(3):100138. doi: 10.1016/j.animal.2020.100138 33573943

[53] Zhang Z, Bi M, Yao H, Fu J, Li S, Xu S. Effect of cold stress on expression of AMPKα–PPARα pathway and inflammation genes. Avian Dis. 2014;58(3):415–26. doi: 10.1637/10763-010814-reg.1 25518437

[54] Shinder D, Luger D, Rusal M, Rzepakovsky V, Bresler V, Yahav S. Early age cold conditioning in broiler chickens (Gallus domesticus): thermotolerance and growth responses. J Ther Biol. 2002;27(6):517–23. doi: 10.1016/s0306-4565(02)00025-6

[55] Nichelmann M, Janke O, Tzschentke B. Efficiency of thermoregulation in precocial avian species during the prenatal period. J Ther Biol. 2001;26(4–5):273–80. doi: 10.1016/s0306-4565(01)00030-4

[56] Subtain SM, Chaudhry ZI, Anjum AA, Maqbool A, Sadique U. Study on pathogenesis of low pathogenic avian influenza virus H9 in broiler chickens. Zoo Soc Pakistan J. n.d.;43(5):999–1008.

[57] Shahid MF, Yaqub T, Tipu MY, Aslam A, Yaqub S, Rahman A, et al. Comparative pathogenic potential of avian influenza A/H9N2 viruses isolated from commercial, backyard and fancy birds. Punjab Univ J Zool. 2019;34(2):143–8. doi: 10.17582/journal.pujz/2019.34.2.143.148

[58] Khantour AE, Houadfi ME, Nassik S, Tligui NS, Mellouli FE, Sikht F-Z, et al. Protective efficacy evaluation of four inactivated commercial vaccines against low pathogenic avian influenza H9N2 virus under experimental conditions in broiler chickens. Avian Dis. 2021;65(3):351–7. doi: 10.1637/aviandiseases-D-21-00015 34427407

[59] Verma AK, Kumar M, Murugkar HV, Nagarajan S, Tosh C, Namdeo P, et al. Experimental infection and in-contact transmission of H9N2 avian influenza virus in crows. Pathogens. 2022;11(3):304. doi: 10.3390/pathogens11030304 35335628 PMC8955285

[60] Kim I-H, Kwon H-J, Kimabce J-H. H9N2 avian influenza virus-induced conjunctivitis model for vaccine efficacy testing. Avian Dis. 2013;57(1):83–7. doi: 10.1637/10240-050712-Reg.1 23678734

[61] Al-Azaway AK, Al-Ajeeli KS, Al-Salihi D. Experimental infection of broilers with H9N2 avian influenza virus local isolate. J Anim Health Prod. 2019;7(4). doi: 10.17582/journal.jahp/2019/7.4.147.157

[62] Aslam R, Aslam A, Tipu Y, Nazir J, Ghafoor A, Fatima S. Histopathological and immunohistochemical studies for the pathogenesis of a low pathogenicity H9 avian influenza virus in experimentally infected commercial broilers. J Anim Plant Sci. 2015;25:45–52.

[63] Swayne DE. Avian Influenza. John Wiley & Sons; 2009.

[64] Tashiro M, Ciborowski P, Klenk HD, Pulverer G, Rott R. Role of *Staphylococcus protease* in the development of influenza pneumonia. Nature. 1987;325(6104):536–7. doi: 10.1038/325536a0 3543690

[65] Tsiouris V, Georgopoulou I, Batzios C, Pappaioannou N, Ducatelle R, Fortomaris P. The effect of cold stress on the pathogenesis of necrotic enteritis in broiler chicks. Avian Pathol. 2015;44(6):430–5. doi: 10.1080/03079457.2015.1083094 26642742

[66] Bóna M, Kiss I, Dénes L, Szilasi A, Mándoki M. Tissue tropism of H9N2 low-pathogenic avian influenza virus in broiler chickens by immunohistochemistry. Animals (Basel). 2023;13(6):1052. doi: 10.3390/ani13061052 36978594 PMC10044543

[67] Awadin W, Said H, Abdin S, El-Sawak AA. Pathological and molecular studies on avian influenza virus (H9N2) in broilers. Asian J Anim Vet Adv. 2018;13(3):232–44. doi: 10.3923/ajava.2018.232.244

[68] Chrzastek K, Lee D-H, Gharaibeh S, Zsak A, Kapczynski DR. Characterization of H9N2 avian influenza viruses from the Middle East demonstrates heterogeneity at amino acid position 226 in the hemagglutinin and potential for transmission to mammals. Virology. 2018;518:195–201. doi: 10.1016/j.virol.2018.02.016 29524835

[69] Jaleel S, Younus M, Idrees A, Arshad M, Khan AU, Ehtisham-Ul-Haque S, et al. Pathological alterations in respiratory system during co-infection with low pathogenic avian influenza virus (H9N2) and *Escherichia coli* in broiler chickens. J Vet Res. 2017;61(3):253–8. doi: 10.1515/jvetres-2017-0035 29978081 PMC5894427

[70] Essalah-Bennani A, Bidoudan Y, Fagrach A, Balil H, Abderrazak EK, Tligui N, et al. Experimental study of the efficacy of three inactivated H9N2 influenza vaccines on broiler flocks. Ger J Vet Res. 2021;1(2):35–45. doi: 10.51585/gjvr.2021.2.0012

[71] Bortolami A, Mazzetto E, Kangethe RT, Wijewardana V, Barbato M, Porfiri L, et al. Protective efficacy of H9N2 avian influenza vaccines inactivated by ionizing radiation methods administered by the parenteral or mucosal routes. Front Vet Sci. 2022;9:916108. doi: 10.3389/fvets.2022.916108 35898545 PMC9309530

