# Supplementary material for: Development of an experimental model using cold stress to assess the pathogenicity of two Moroccan AI H9N2 isolates from 2016 and 2022 in commercial broiler chickens
Source: PLoS One. 2025 Apr 4;20(4):e0320666. doi: 10.1371/journal.pone.0320666 (PMC11970702; doi:10.1371/journal.pone.0320666)
Supplement: S2 Appendix — (PDF) [file pone.0320666.s002.pdf]

S2 Appendix. Body weight gains during the different periods of the experiment.

BWG (g)	Experimental groups				P values
	Group A	Group B	Group C	Group D	
**D1 to D21**	664 ±16[a]	653 ±14[a]	658 ±14[a]	662 ±16[a]	0,152>0,05
**D22 to D35**	976 ±87 [b]	914 ±62 [b]	853 ±235 [b]	1003 ±60 [b]	0,85>0,05
**D1 to D35**	1728 ±101[c]	1773 ±74 [c]	1789 ±197 [c]	1800 ±62 [c]	0,978>0,05

BWG = body weight gain
Similar superscript letters in the same row indicates no significance differences (P>0.05.
