# Supplementary material for: Development of an experimental model using cold stress to assess the pathogenicity of two Moroccan AI H9N2 isolates from 2016 and 2022 in commercial broiler chickens
Source: PLoS One. 2025 Apr 4;20(4):e0320666. doi: 10.1371/journal.pone.0320666 (PMC11970702; doi:10.1371/journal.pone.0320666)
Supplement: S3 Appendix — (PDF) [file pone.0320666.s003.pdf]

S3 Appendix. Clinical mean respiratory and ocular symptom scores.

Factors	Days post-infection	Groups A (Challenged with 2016 strain)	B (Challenged with 2022 strain)	C (Received only cold stress)	D (Negative control)
Median respiratory sign scores	**4**	1,23 ±0,19^{a}	2,5 ±0,93^{b}	0,9 ±0,23^{c}	0^{c}
	**6**	2,36 ±0,17^{a}	2,61 ±0,12^{a}	0,5 ±0,^{19c}	0^{c}
	**8**	1,86 ±0,14^{a}	2,39 ±0,12^{b}	0^{c}	0^{c}
	**10**	0,93 ±0,73^{a}	2,17 ±0,51^{b}	0^{c}	0^{c}
Median ocular sign scores	**4**	0,41 ±0,18^{b}	1,35 ±0,2^{a}	0^{b}	0^{b}
	**6**	1,36±0.11^{a}	1,56±0,45^{a}	0^{b}	0^{b}
	**8**	1,64±0,21^{a}	2,11±0,58^{a}	0^{b}	0^{b}
	**10**	1,07 ±0,22^{a}	1,89 ±0,18^{b}	0^{c}	0^{c}

Different superscript letters in the same row indicate a significant difference ($P<0.05$).
