# Supplementary material for: Development of an experimental model using cold stress to assess the pathogenicity of two Moroccan AI H9N2 isolates from 2016 and 2022 in commercial broiler chickens
Source: PLoS One. 2025 Apr 4;20(4):e0320666. doi: 10.1371/journal.pone.0320666 (PMC11970702; doi:10.1371/journal.pone.0320666)
Supplement: S4 Appendix — (PDF) [file pone.0320666.s004.pdf]

S4 Appendix. Gross pathological lesions observed on the respiratory tract at 5, 10 and 15 dpi.

Macroscopic lesions	Days post-infection	Groups A	B	C	D	P- Value
Congestive tracheitis	5	100% (10/10)^{a}	100% (10/10)^{a}	50%(5/10)^{b}	0% (0/10)^{c}	*0.001<0.05*
	10	70% (7/10)^{a}	80% (8/10)^{a}	0% (0/10)^{b}	0% (0/10)^{b}	*0.001<0.05*
	15	40% (4/10)^{a}	71.4% (5/7)^{b}	0% (0/10)^{c}	0% (0/10)^{c}	*0.002<0.05*
Fibrinous tracheitis	5	50% (5/10)^{a}	60% (6/10)^{a}	0% (0/10)^{ab}	0% (0/10)^{b}	*0.025<0.05*
	10	10% (1/10)^{a}	10% (1/10)^{a}	0% (0/10)^{a}	0% (0/10)^{a}	*0.45>0.05*
	15	0% (0/10)^{a}	0% (0/7)^{a}	0% (0/10)^{a}	0% (0/10)^{a}	*0.58>0.05*
Fibrinous plugs	5	10% (1/10)^{a}	40% (4/10)^{b}	0% (0/10)^{ab}	0% (0/10)^{a}	*0.016<0.05*
	10	0% (0/10)^{a}	0% (0/10)^{a}	0% (0/10)^{a}	0% (0/10)^{a}	*0.573>0.05*
	15	0% (0/10)^{a}	0% (0/7)^{a}	0% (0/10)^{a}	0% (0/10)^{a}	*0.58>0.05*
Congestive pneumonia	5	30% (3/10)^{a}	100% (10/10)^{b}	0% (0/10)^{a}	0% (0/10)^{a}	*0.001<0.05*
	10	20% (2/10)^{a}	60% (6/10)^{b}	0% (0/10)^{c}	0% (0/10)^{c}	*0.007<0.05*
	15	10% (1/10)^{a}	75% (6/8)^{b}	0% (0/10)^{c}	0% (0/10)^{c}	*0.001<0.05*
Fibrinous pneumonia	5	0% (0/10)^{a}	10% (1/10)^{a}	0% (0/10)^{a}	0% (0/10)^{a}	*0.001<0.05*
	10	0% (0/10)^{a}	10% (1/10)^{b}	0% (0/10)^{a}	0% (0/10)^{a}	*0.001<0.05*
	15	0% (0/10)^{a}	0% (0/7)^{a}	0% (0/10)^{a}	0% (0/10)^{a}	*0.58>0.05*
Fibrinous airosaculitis	5	20% (2/10)^{a}	20% (2/10)^{a}	0% (0/10)^{a}	0% (0/10)^{a}	*0.251>0.05*
	10	10% (1/10)^{a}	40% (4/10)^{b}	0% (0/10)^{a}	0% (0/10)^{a}	*0.004<0.05*
	15	0% (0/10)^{a}	0% (0/7)^{a}	0% (0/10)^{a}	0% (0/10)^{a}	*0.58>0.05*
Fibrinous sinusitis	5	20% (2/10)^{a}	10% (1/10)^{a}	0% (0/10)^{a}	0% (0/10)^{a}	*0.29>0.05*
	10	10% (1/10)^{a}	20% (2/10)^{b}	0% (0/10)^{a}	0% (0/10)^{a}	*0.024<0.05*
	15	0% (0/10)^{a}	0% (0/7)^{a}	0% (0/10)^{a}	0% (0/10)^{a}	*0.58>0.05*

Different superscript letters in the same row indicate a significant difference (P<0.05).
